# Key residues in SARS-CoV-2 NSP3 hypervariable region are necessary to modulate early stress granule activity

**DOI:** 10.1128/jvi.02006-25

**Published:** 2026-01-14

**Authors:** R. Elias Alvarado, Kumari G. Lokugamage, Dimitriya Garvanska, Leah K. Estes, Yani Ahearn, Alyssa M. McLeland, Arian Moayyed, Jennifer Chen, Blanca Lopez Mendez, Jessica A. Plante, Kenneth S. Plante, Bryan A. Johnson, Jakob Nilsson, Vineet D. Menachery

**Affiliations:** 1 Department of Microbiology and Immunology, University of Texas Medical Branch 547647, Galveston, Texas, USA; 2 Human Pathophysiology and Translational Medicine, University of Texas Medical Branch 12338, Galveston, Texas, USA; 3 Institute for Translational Sciences, University of Texas Medical Branch 461038, Galveston, Texas, USA; 4 Department of Pediatrics, Emory University School of Medicine 12239, Atlanta, Georgia, USA; 5 Novo Nordisk Foundation Center for Protein Research, Faculty of Health and Medical Sciences, University of Copenhagen 53139, Copenhagen, Denmark; 6 Institute for Human Infection and Immunity, University of Texas Medical Branch 12338, Galveston, Texas, USA; 7 Emory Vaccine Center, Emory University 1371, Atlanta, Georgia, USA; Dartmouth College Geisel School of Medicine, Hanover, New Hampshire, USA

**Keywords:** SARS-CoV-2, coronavirus, NSP3, FXR1, nucleocapsid, stress granules

## Abstract

Antagonism of the host responses that limits viral replication is critical to the success of infection. Recently, we identified that the hypervariable region (HVR) of SARS-CoV-2 NSP3 binds to FXR1 and disrupts stress granule formation during the early stages of infection. Despite variation across the rest of the HVR, a 20-amino acid region, highly conserved in the Sarbecovirus family, is required for NSP3-FXR1 binding, but the critical residues remained unresolved. In this study, we explore the individual residues in NSP3 driving FXR1 binding and determine their impact on viral replication, pathogenesis, and stress granule formation. Our results indicate that the tyrosine at position 138 (Y138) and a phenylalanine at position 145 (F145) are required for FXR1 binding and affinity. Using reverse genetics, we showed that mutating NSP3 Y138A/F145A (YF mutant) reduced viral replication *in vitro* and *in vivo*. Importantly, we demonstrate that attenuation is not due to differential type I interferon responses but rather loss of stress granule control by the NSP3 mutant as compared to wild type. Together, our findings demonstrate the importance of Y138 and F145 within the NSP3-HVR in regulating stress granule formation at the early times post-infection.

**IMPORTANCE:**

Stress granules play a key role in host-antiviral defenses, and viruses have developed strategies to antagonize their activity. For SARS-CoV-2, the virus has two proteins that antagonize stress granules, with NSP3 acting early and nucleocapsid acting at late times. Here, we show that key NSP3 residues Y138 and F145, conserved across the Sarbecovirus family, are necessary to bind FXR1 and disrupt its activity in stress granule formation. Mutating these residues results in attenuation of SARS-CoV-2 replication and induces stress granule formation at early times post-infection. These results show the importance of these NSP3 residues in disrupting stress granule formation early and highlight multiple approaches SARS-CoV-2 uses to antagonize stress granule activation.

## INTRODUCTION

SARS-CoV-2 is the causative agent of COVID-19 and is responsible for an unprecedented six million deaths worldwide (1). Despite our incredible strides in medicine and research, our understanding of how SARS-CoV-2 induces disease and evades immunity remains limited. To solve this problem, future research will require a molecular understanding of events driving pathogenesis and viral replication.

SARS-CoV-2 is a positive-sense, single-stranded RNA virus with a genome size approximately 30 kb (2). The viral genome contains two open reading frames (ORFs) that encode two polyproteins: polyprotein 1A and 1AB. After translation, the polyproteins are cleaved into their individual non-structural protein (NSP) components (3). Overall, the SARS-CoV-2 genome encodes 16 non-structural proteins (NSP1–16), 4 structural proteins, and 9 accessory proteins. Extensive research has shown that NSPs play critical roles in supporting viral replication while simultaneously evading innate immune responses. Among them, NSP3 is the largest protein that contains several conserved and non-conserved domains that are exclusive to *Sarbecovirus* (4, 5). At the N-terminus of NSP3 lies a unique region rich in Glu/Asp amino acids termed the hypervariable region (HVR) (6). Despite being a region that exists in all CoVs, the function of the HVR has yet to be determined. Being an intrinsically disordered region, the HVR harbors short (3–10 residues) fixed amino acid sequences called short linear motifs that can serve as important sites for protein-protein interactions (7). Therefore, the HVR has the potential to serve as a hub for binding to host cellular factors during infection.

Foreign stressors, including virus infections, trigger cells to temporarily halt protein translation to focus efforts on survival and return to homeostatic conditions. Stress granule (SG) formation is one response that occurs in the context of host translation arrest and is frequently observed during viral infections (8). SGs, membrane-less cytoplasmic structures, are large focal complexes composed of untranslated mRNAs, protein translation factors, and RNA-binding proteins (RBPs) (9, 10). Upon activation, SG components like G3BP1, FXR proteins, and TIA-1 form condensates through liquid-liquid phase separation that is driven by multivalent interactions between the granule constituents (9, 11). When these liquid droplets form, untranslated mRNA and protein translation factors are sequestered, impairing translation. Once the stressor is gone, SGs disassemble and release these factors, allowing translation to proceed. Importantly, viral replication is dependent on the host translational machinery, and thus, SGs play a role in host-antiviral responses. Therefore, viruses have evolved ways to inhibit SGs by targeting key SG components, thereby disrupting the formation or facilitating disassembly of SGs during infection.

Among the key SG components, G3BP1 is an RNA-binding protein that is the central regulator for SG formation (12, 13). G3BP1 plays a critical role in the dynamics of SGs via its contributions to liquid-liquid phase separation (14). Importantly, G3BP1 is a common target of viral antagonism, which often serves to disrupt SG activity following infection (15–19). Our group and others have identified SARS-CoV-2 nucleocapsid as binding to G3BP1 and disrupting SG activity following infection (20). However, other RBPs, including the FXR family of proteins, have been implicated in SG formation and can be targeted by viruses during infection (21–24). Associated with Fragile X syndrome and other genetic diseases (25), FXR proteins have a wide range of regulatory functions, including formation of SGs. Utilizing the intrinsically disordered K-homology 2 domain, FXR proteins can undergo liquid-liquid phase separation, which likely contributes to their role in SG formation (26, 27). Importantly, viruses including Old World (EEEV) and New World (VEEV) alphaviruses, adenovirus, and, recently, coronaviruses have also been shown to antagonize FXR function during infection (23, 28, 29). Given the reports of antagonism across multiple virus families, these results argue that there is a critical role for FXR proteins in SG activities and antiviral function.

Recently, our group identified that the HVR in NSP3 of SARS-CoV-2 acts as a hub to bind and hijack FXR1 away from SGs during early stages of infection (30). We show that cells expressing NSP3-HVR hijack FXR1 away from SG but do not affect the overall number of SGs formed. Despite these findings in protein-expressing cells, we cannot exclude that FXR1 and NSP3 interactions are important during actual viral infection. To further understand if the HVR of NSP3 is important for the regulation of SGs, we sought to refine our NSP3 viral mutant and further define the role of FXR1 during infection. Here, we found that tyrosine at position 138 (Y138) and phenylalanine at position 145 (F145) are key residues that are important for FXR1 binding and affinity. Using our reverse genetics system, we found that SARS-CoV-2 mutants incapable of binding to FXR1 (YF mutant) show reduced viral replication *in vitro* and reduced viral load *in vivo* and that this attenuation is not driven by type I interferon (IFN) responses. Mechanistically, we show that preventing FXR1 binding to the HVR is important for recovering SG formation and that FXR1 acts as a proviral factor to support efficient viral replication. Our findings reveal that the NSP3-HVR is important for regulating SG formation at the early phase of infection to facilitate viral replication.

## RESULTS

### NSP3 F145 and Y138 are key to binding to FXR1

**Fig 1:**
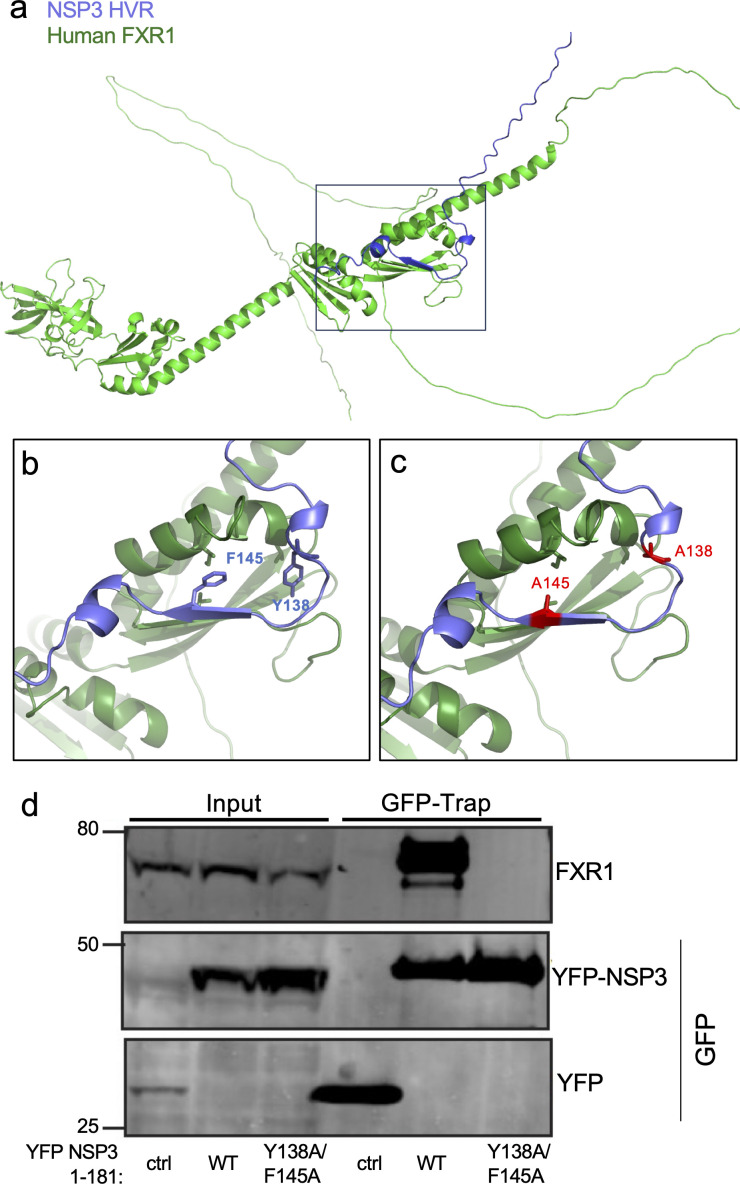
NSP3 mutations at Y138 and F145 disrupt peptide interactions with FXR1. (**a**) AlphaFold model of the FXR1-NSP3 complex interactions. (**b**) Purple indicates wild-type NSP3 aromatic residues, (**c**) while red indicates residues changed to alanine. (**d**) The indicated NSP3 fragments were fused to YFP, expressed, and immunoprecipitated from HeLa cells to examine binding to FXR1 by immunoblotting. Represented are two biological replicates.

**Fig 2:**
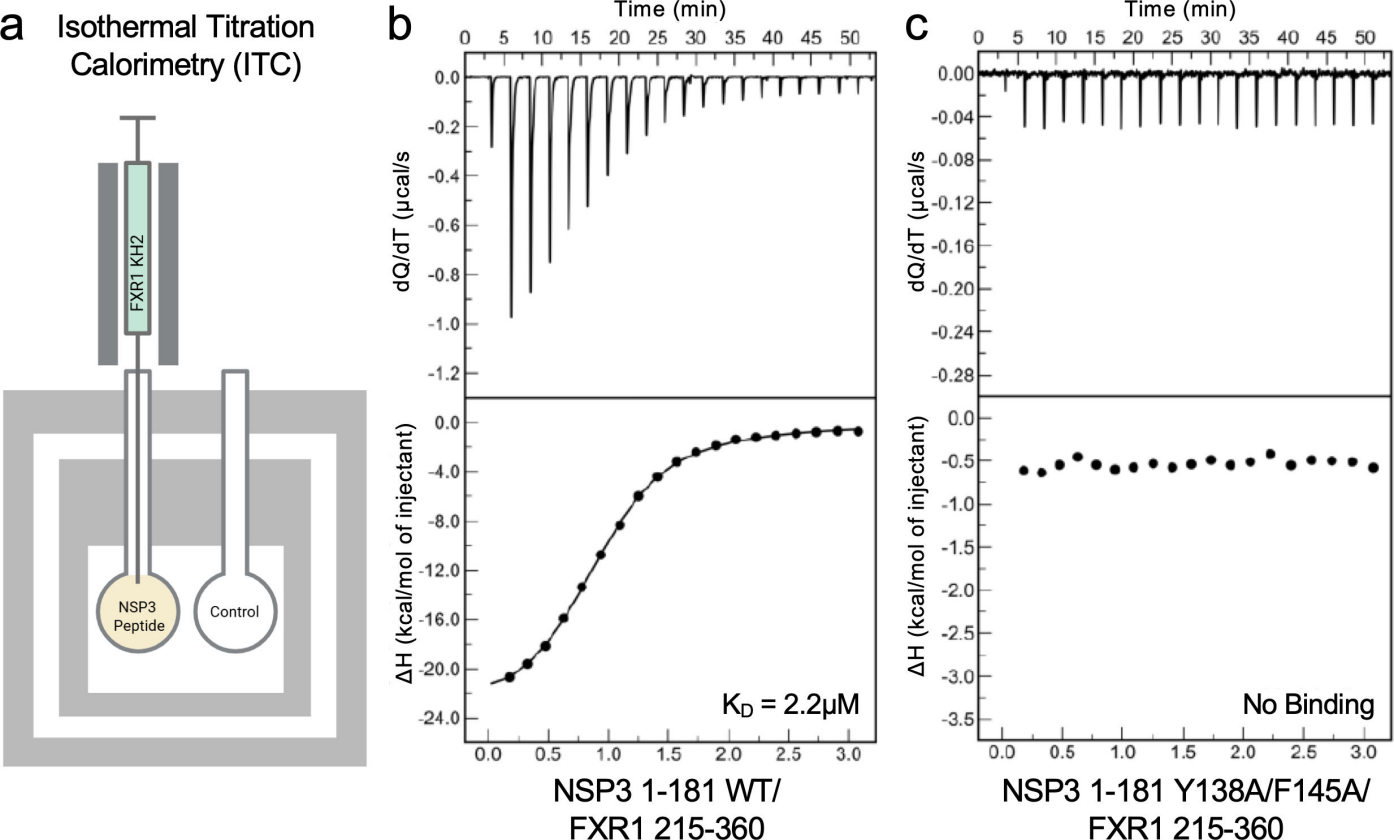
YF mutations ablate binding between NSP3 and FXR1. (**a**) Schematic of isothermal titration with injection of WT or mutant NSP3 peptide into chamber with human FXR1 peptide. A calorimeter measures the change in heat as the NSP3 peptide is titrated to calculate binding affinity (*K*_*D*_). Figure made using BioRender. (**b and c**) Isothermal titration results between the WT NSP3 peptide (**b**) and the NSP3-YF mutant peptide (**c**) show ablation of binding in the YF mutant compared to WT NSP3.

Our prior studies showed that SARS-CoV-2 NSP3 amino acid regions 129–149 interact with human FXR1 and that two SARS-CoV-2 alanine scanning mutants covering the 20 AA region were attenuated (30). The alanine scanning results revealed that multiple residues are responsible for the interaction. Additionally, a subsequent peptide array suggested a critical role for Y138 and F145 (30). Examining the AlphaFold-based structure (Fig. 1a), we found that the binding interface between FXR1 and NSP3 shows an interaction between FXR1 I304 and NSP3 F145 (Fig. 1b). While Y138 is not directly involved in the binding interface, the aromatic residue may offer stability to the loop, allowing the FXR1/NSP3 interaction. To verify the role of these amino acids in binding, we generated a wild type (WT) and Y138A/F145A mutant YFP-NSP3 fusion peptide (NSP3 1-181AA) (Fig. 1c). The YFP-NSP3 fusion proteins were transfected, and lysates were cleared and incubated with GFP-trap beads; following washing, samples were processed by Western blot for FXR1 and GFP (Fig. 1d). As expected, GFP-trap lysates resulted in the pull-down of FXR1 with WT YFP-NSP3 peptide; however, mutations Y138A/F145A ablated NSP3 binding to FXR1, demonstrating the importance of both residues for the FXR1/NSP3 interaction. We subsequently used isothermal titration to examine the molecular interactions between WT and mutant NSP3 peptide with FXR1 (Fig. 2a). Injecting WT YFP-NSP3 peptide, we observed robust binding (2.2 μM) to FXR1 (Fig. 2b); these results were consistent with our previous results (30). In contrast, the mutation at Y138A/F145A in the NSP3 mutant peptide ablated binding to FXR1 (Fig. 2c). Together, these findings demonstrate that both Y138 and F145 in SARS-CoV-2 NSP3 are necessary for the FXR1 binding, and their absence ablates the interaction.

### Disrupting FXR1 binding to NSP3-HVR reduces viral replication *in vitro*

**Fig 3:**
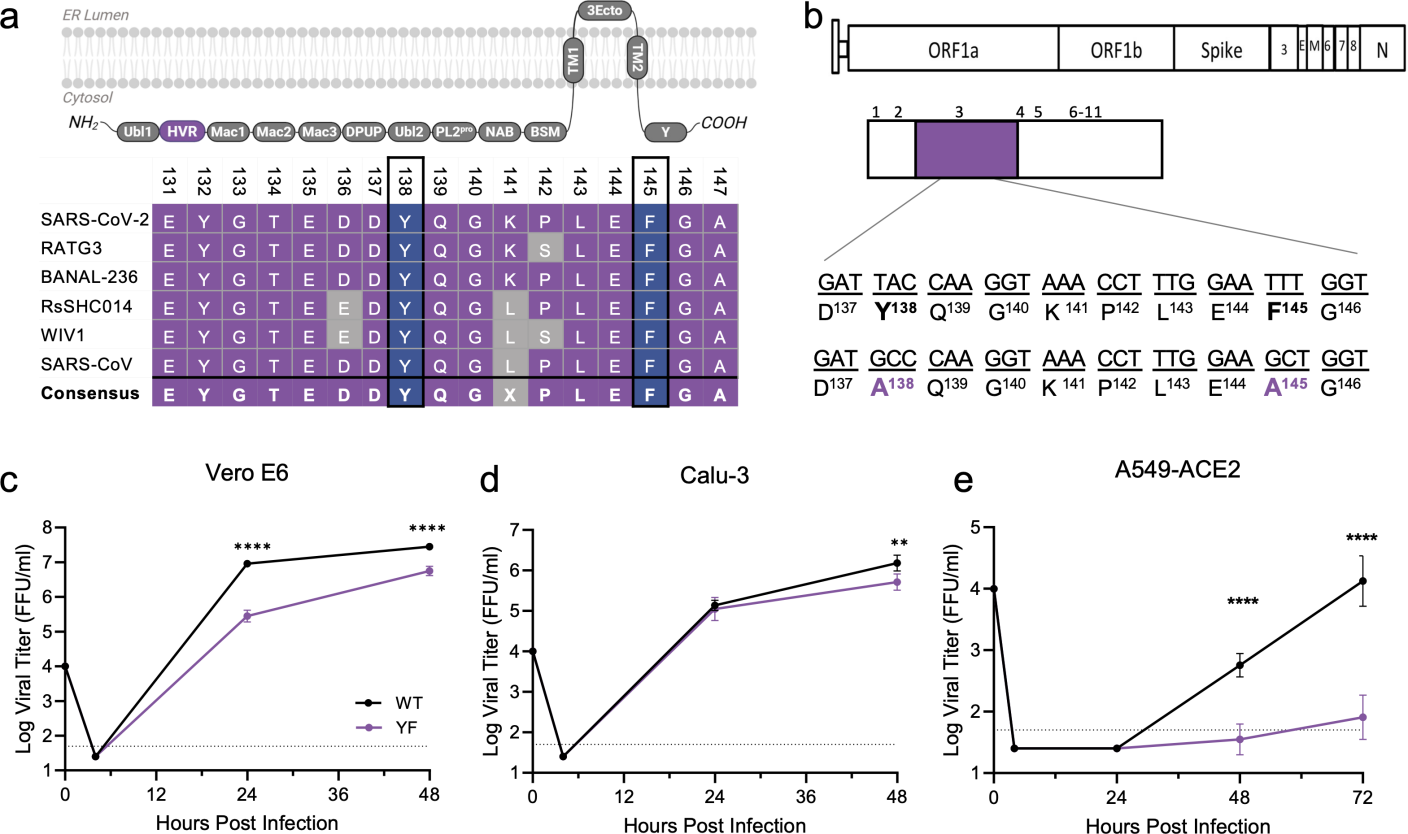
SARS-CoV-2 YF mutant has attenuated viral replication *in vitro*. (**a**) Schematic of the NSP3 domains including the hypervariable region (HVR, purple) made using BioRender. Sequence alignment of *Sarbecovirus* members NSP3-HVR shows conservation of Y138 and F145 across virus family. (**b**) Schematic of SARS-CoV-2 NSP3 with YF mutations to alanine (purple) introduced in the Washington 1 strain. (**c–e**) Viral titer from Vero E6 (**c**), Calu3 (**d**), or A549-ACE2. (**e**) Cells infected with WT (black) or YF (purple) SARS-CoV-2 at a multiplicity of infection of 0.01 (*n* = 6 from two experiments, each with three biological replicates). Data are representative of mean ± SD. Statistical analysis measured by two-tailed Student’s *t*-test. **P ≤ 0.01, ****P < 0.0001.

Y138 and F145 are both harbored within the HVR of NSP3, the largest viral protein produced during infection (Fig. 3a). To determine if these residues are conserved, we conducted a sequence alignment of NSP3-HVR from across *Sarbecovirus* members (Fig. 3b). Both Y138 and F145 were conserved in all *Sarbecoviruses* surveyed; however, similar residues or motifs could not be aligned from the NSP3 HVR of CoVs from the other alpha and beta CoV families. Interestingly, bat-derived CoVs BANAL-236 and RATG3, the most SARS-CoV-2 similar sequences, retain all 20 residues in the FXR1 binding motif. In contrast, the bat CoVs RsSHC014 and WIV1, more closely related to SARS-CoV, have six to seven residue differences compared to the SARS-CoV-2 consensus. Overall, despite the variation in the NSP3 HVR, Y138 and F145 are completely conserved across the entire Sarbecovirus family.

Having demonstrated that residues Y138 and F145 are necessary for binding with FXR1 (Fig. 1 and 2), we next evaluated the effects of disrupting this interaction during authentic viral infection. Here, we generated a SARS-CoV-2 NSP3 mutant that substitutes Y138 and F145 to alanine (YF) into the WT WA-1 backbone using our reverse genetics system (31, 32) (Fig. 3c). Introducing the YF mutations caused no deficits in viral stock titer or changes in plaque morphology. We next compared the viral replication kinetics of the YF mutant to WT across different cell lines using a low multiplicity of infection (MOI). We started with the infection of Vero E6 cells, an African green monkey kidney cell line that lacks competent type I IFN responses (Fig. 3d). Following infection, the YF mutant had significant attenuation at both 24 and 48 h post-infection (hpi). We next evaluated the YF mutant in Calu3-2B4 cells, a human bronchial epithelial cell line with robust type I interferon responses (33). Following low MOI (0.01) infection, the YF mutant replicated to similar viral titers as WT at 24 hpi (Fig. 3e). By 48 h, the YF mutant had a significant, but modest reduction in viral yields as compared to WT control. These results were in contrast to Vero E6 results and demonstrated that attenuation of the YF mutant varied across different cell lines *in vitro*.

While we often observe a discrepancy between Vero E6 and Calu3 cells in regard to SARS-CoV-2 mutant replication, these differences often correlate with either the expression of TMPRSS2 or type I IFN response, both absent in Vero cells (34, 35). With the opposite pattern, it argued that attenuation may be driven by different factors including stress granule activity or control. To further examine the role of SGs, we examined A549-hACE2 cells, a lung adenocarcinoma cell line that is transduced to stably express the human ACE2 receptor. Prior studies found robust SG responses are induced in A549 cells following virus infection, and numerous SG assays had been evaluated using these cells (36). Following infection, A549-hACE2 cells had significant attenuation of the YF mutant as compared to control (Fig. 3f). While no detectable virus was observed at 24 h in either sample, at both 48 and 72 h, the WT virus far exceeded the mutant in terms of viral load. These results confirmed attenuation of the YF mutant and argue that SG responses drive differences in viral load following infection.

### SARS-CoV-2 YF mutant attenuates replication *in vivo*

**Fig 4:**
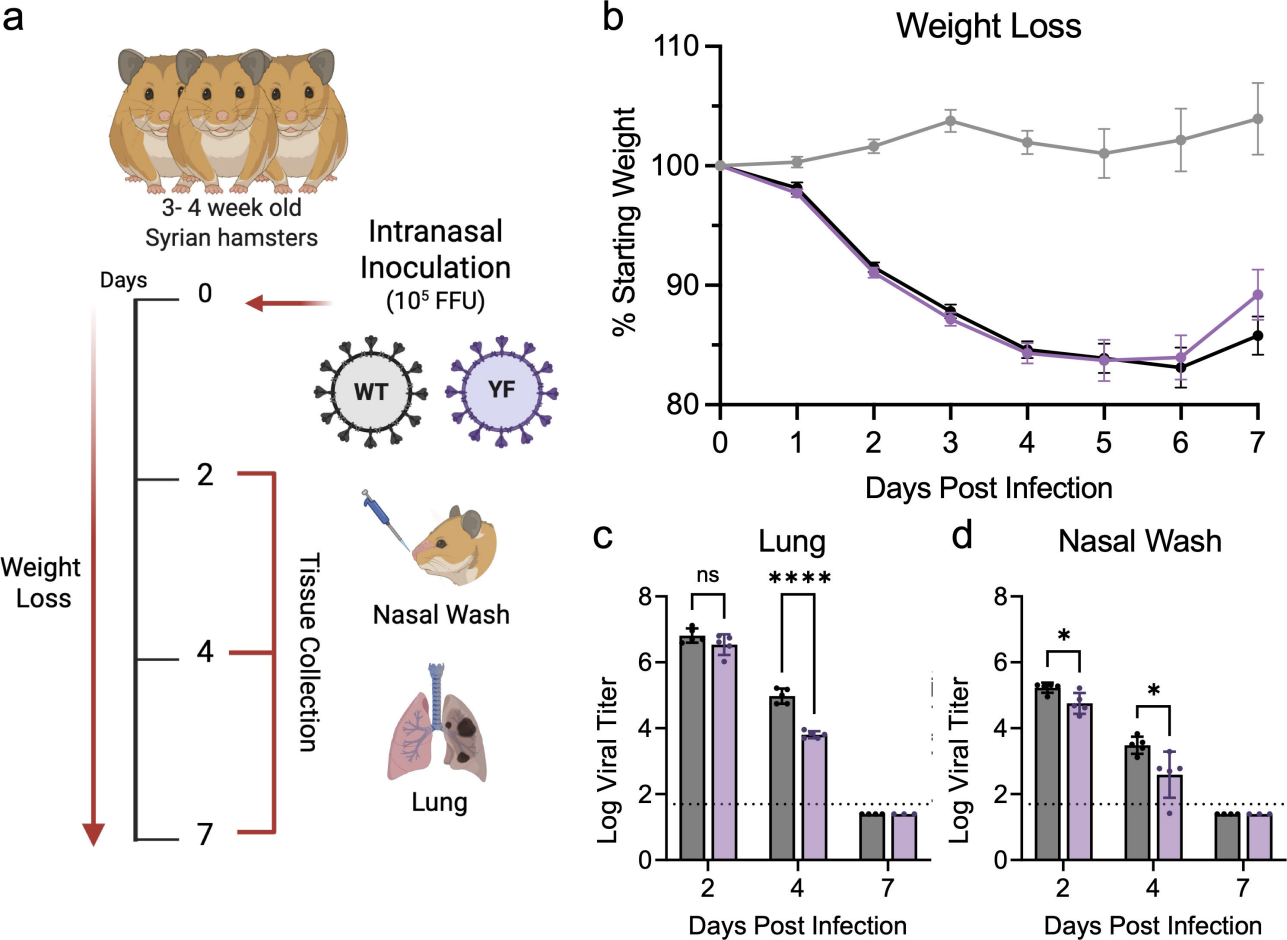
The YF mutant has modest attenuation *in vivo*. (**a**) Schematic of golden Syrian hamster infection with WA-1 (WT) or YF mutant (purple) made using BioRender. Three- to four-week-old male hamsters were intranasally inoculated with 10^5^ FFU of WT or YF and monitored daily for signs of disease and weight loss (**b**) over 7 days. Infectious titers were measured in the lungs (**c**) and nasal wash (**d**) on 2, 4, and 7 dpi. Each dot represents an infected animal. The dotted lines represent the assay limit of detection. Statistical significance was determined by two-tailed Student’s t-test. *P ≤ 0.05, ****P < 0.0001.

We next evaluated whether loss of FXR1 binding also attenuates viral replication *in vivo* using the golden Syrian hamster model for infection (Fig. 4a) (37–39). Briefly, 3- to 4-week-old male hamsters were intranasally challenged with 10^5^ FFU of either SARS-CoV-2 WT or YF (Fig. 4a) and monitored for weight loss and disease over 7 days. At 2, 4, and 7 days post-infection (dpi), animals were nasal washed and euthanized for tissue collection to determine viral load changes. Similar to our previous work (30), YF-infected hamsters exhibited weight loss and disease similar to those of the WT cohort (Fig. 4b). However, viral load in the upper and lower respiratory tracts revealed significant differences. On days 2 and 4 dpi, viral titers from nasal washes were lower in YF-infected animals compared to WT (Fig. 4c and d). At 4 dpi, YF-infected hamsters displayed significantly lower titers compared to the WT-infected group (Fig. 4c and d). No virus was detectable at 7 dpi in both specimens. These results reflect our *in vitro* findings and show that disrupting FXR1 binding with two residues in the NSP3-HVR is critical for attenuating replication.

### Attenuation of the YF mutant is not due to augmented interferon responses

**Fig 5:**
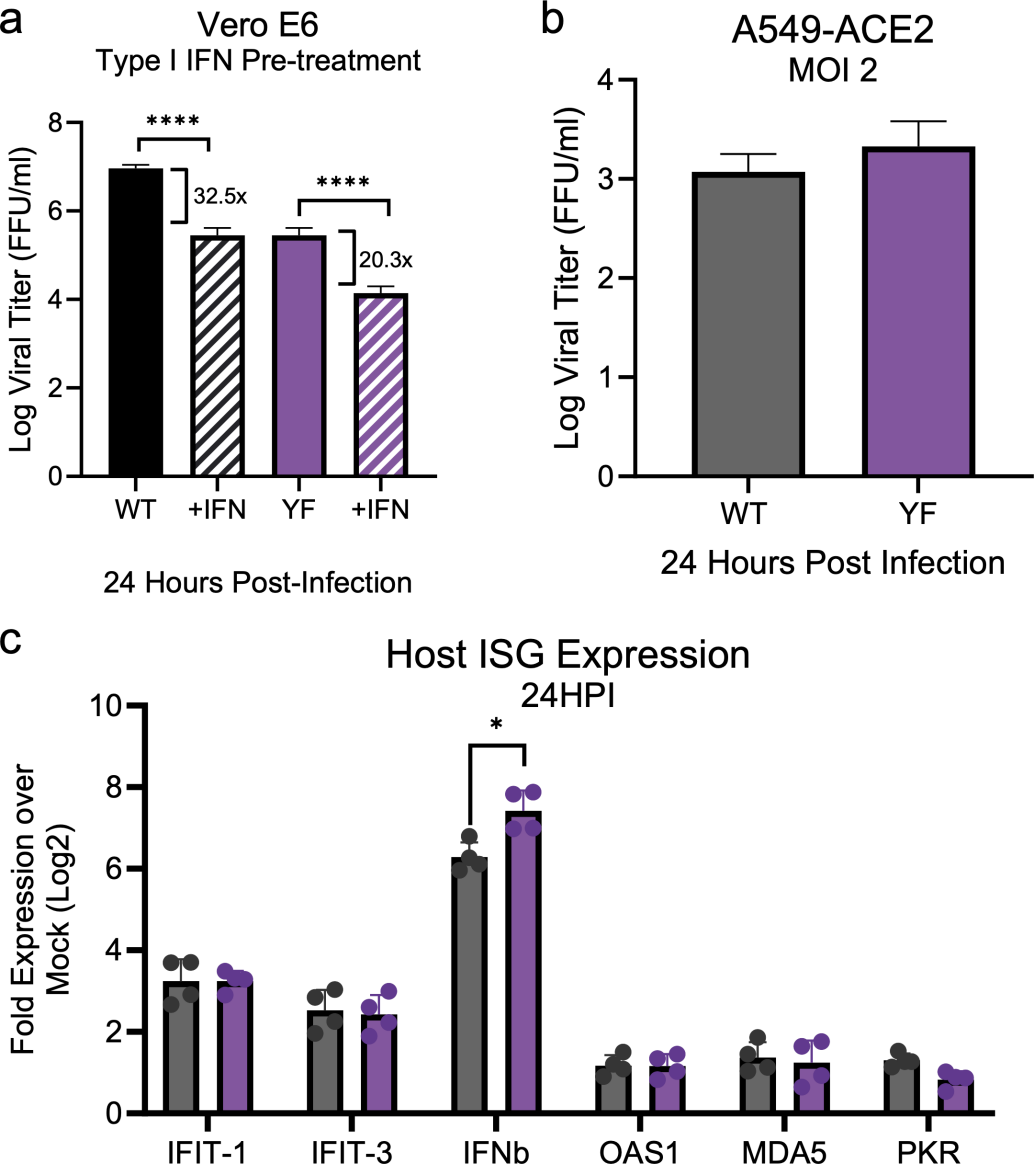
Decreased YF mutant replication is not driven by innate immune responses. (**a**) Vero E6 cells were pretreated with 100 U of recombinant type I IFN (hashed bar) or mock (solid bar) for 16 h prior to infection. Cells were subsequently infected with either SARS-CoV-2 WT (black) or YF mutant (purple) at an MOI of 0.01. Viral titers were measured at 24 hpi. The fold change relative to mock control is shown. (**b**) A549-ACE2 cells were mock, WT, or YF infected at an MOI of 1 for 24 h. (**c**) Fold expression of interferon stimulated genes (ISGs) relative to mock infected sample. At 24 hpi, total RNA was collected and host mRNA expression was quantified by RT-qPCR. *C*_*t*_ values were normalized to human GAPDH and expressed as fold change (log_2_) of expression of indicated genes. Each dot represents a technical replicate, with the means for each target displayed ± SD (error bars). Statistical significance was determined by two-tailed Student’s *t*-test. *P ≤ 0.05, **P ≤ 0.01, ***P ≤ 0.001, ****P < 0.0001.

Both stress granule induction and type I interferon responses are antiviral responses induced by the cell (40–42). While NSP3 binding to FXR1 links to stress granule formation, its implication for type I interferon and related responses is less clear. Here, we evaluated if the YF mutation sensitizes SARS-CoV-2 to type I IFN responses. A key difference among the cell lines used is that Calu3 and A549 cells are type I IFN competent (33). Vero E6 cells cannot produce type I IFN but can respond to exogenous treatment (43). Therefore, we investigated the effect of type I IFN on YF mutant replication. We pretreated Vero E6 cells with 100 U of recombinant type I IFN (IFN-α) 16 h prior to infection and compared samples against mock (PBS) pretreated controls. Similar to previous work, the WT virus had only modest sensitivity to type I IFN pretreatment and resulted in a modest ~log reduction in viral titer at 24 h (Fig. 5a). Similarly, the YF mutant also had modest sensitivity to type I IFN pretreatment; viral titer reduced ~1 log at 24 hpi. In contrast to type I IFN-sensitive SARS-CoV-2 mutants that show >3 log attenuation following IFN pretreatment (44), the similarity in reduction between the WT and YF mutant indicates that type I IFN responses are not the main driver of attenuation.

To further examine if the attenuation of the YF mutant is driven by host innate immune pathways, we investigated the induction of type I and interferon-stimulated genes. The largest differences between WT and YF mutants in viral titer were observed in A549-ACE2 cells which have intact SG and type I IFN responses (Fig. 3f) (45, 46). Here, we sought to equalize the viral load by infecting at a high MOI of 2 to ensure maximum infection and compare host expression differences between WT and mutant virus. Following infection, we found that viral replication was equivalent between WT and YF mutant 24 hpi (Fig. 5b). We subsequently examined the expression of innate immune response genes including type I IFN (*IFNB1*) and several IFN-stimulated genes (*IFIT1*, *MDA5*, *OAS1b*, *PKR*, and *IFIT3*) (Fig. 5c). While we note a modest increase in *IFNB1* expression in the mutant, the other ISGs are largely similar, suggesting that differential innate immune responses are not responsible for attenuation of the YF mutant.

### Stress granule formation is observed in YF mutant-infected cell lines early during infection

**Fig 6:**
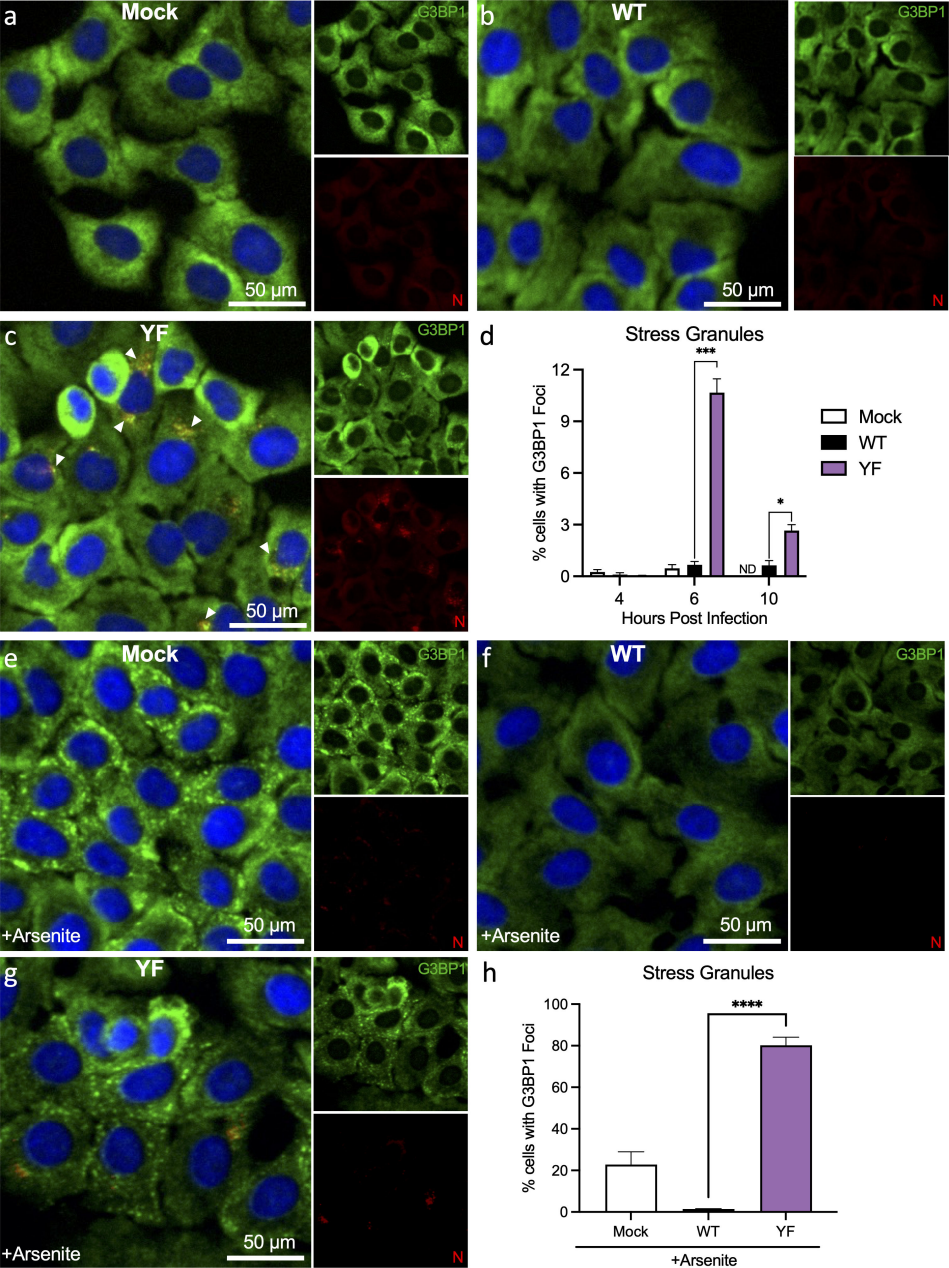
Stress granule formation is restored in YF mutant-infected A549-ACE2 cells during early infection. A549-ACE2 cells were infected with WT (**b**) or YF mutant (**c**) at an MOI of 1 for 4, 6, and 10 hpi. Cells treated with PBS (**a**) were used as a control. G3BP1 (green), nucleocapsid (red), and nuclei (blue) were labeled to visualize by immunofluorescence imaging. (**a–c**) Images at 6 hpi are represented. (**d**) The total percentage of cells with G3BP1+ foci (indicates SGs) was calculated using Fiji (ImageJ) software. (**e–h**) A549-ACE2 cells were infected with WT (**f**) or YF mutant (**g**) at an MOI of 1 for 6 hpi. Cells treated with PBS (**e**) were used as a control. Thirty minutes prior to 6 h time point, cells were treated with 1 mM sodium arsenite to induce SG formation. (**h**) The total percentage of cells with G3BP1+ foci at 6 hpi was calculated. All quantitative data are shown as mean ± SD. Statistical analysis measured by two-tailed Student’s *t*-test. *P ≤ 0.05, ***P ≤ 0.001, ****P < 0.0001. ND, detected.

Our prior studies demonstrated that SARS-CoV-2 NSP3 disrupts incorporation of FXR1 into SGs by competing with UBAP2L in a virus-free protein expression system (30). However, it is unclear if SG induction occurs in the context of infection with the SARS-CoV-2 YF mutant. Complicating this analysis, the N protein of SARS-CoV-2 has been shown by our group to be a potent SG inhibitor, though we predict its effect occurs primarily at late time points (20). Therefore, to evaluate SG dynamics following WT and YF mutant infection, we infected A549-ACE2 at 4, 6, and 10 hpi at an MOI of 1 and analyzed SGs by immunofluorescence. Visualizing infected cells, we stained for SARS-CoV-2 nucleocapsid (red), G3BP1 (green), and nuclei (Hoechst) and observed varying motifs at 6 hpi (Fig. 6a through c). We first examined the percentage of cells that had the formation of G3BP1 foci and found no significant induction in any of the conditions at 4 hpi (Fig. 6d). However, by 6 hpi, we observed that ~11% of cells in the YF-infected group formed at least one SG as measured by G3BP1 foci (Fig. 6c and d). In contrast, <1% of cells were observed to have G3BP1 foci in WT- and mock-infected cells at 6 hpi (Fig. 6a, b, and d). Notably, we found low nucleocapsid staining in WT, but N appears to aggregate at the SG in the YF mutant as seen in the merge (Fig. 6C, white arrowheads). These results correspond with SG levels peaking at 6 hpi in YF infection, but by 10 hpi, SG levels had reduced significantly to 2.7% in the YF mutant, although still elevated relative to 0.6% in WT. These results indicate that N protein has a potent impact on SGs at later times post-infection.

Given the low amount of SG formation in standard infection (~10%), we next determined if the YF mutant could inhibit SG formation if actively induced. To test this possibility, we treated cells with sodium arsenite, a chemical inducer of oxidative stress and SG formation. Mock cells treated with sodium arsenite induced SGs as measured by punctate G3BP1 foci (Fig. 6e and h). In contrast, WT-infected cells actively inhibited SG formation and showed no evidence of G3BP1 foci (Fig. 6f and h). Importantly, YF-infected cells had roughly 80% of cells showing G3BP1 foci (Fig. 6g and h). These results demonstrate that the YF mutant not only fails to inhibit SG formation at the early stage of infection but also augments SG formation beyond arsenite treatment alone. Overall, our results indicate that the loss of NSP3/FXR1 binding in the YF mutant induces a significant increase in SG-positive cells by 6 hpi. While these dissipate at late times, likely due to N activity, it highlights the role of NSP3 and binding to FXR1 in disrupting SG formations early following infection.

## DISCUSSION

Our previous studies revealed a functional role for the SARS-CoV-2 HVR in NSP3 for binding FXR1 and disrupting SG processes. Here, we extended this work by identifying residues Y138 and F145 in the NSP3 HVR as required for binding with FXR1. Subsequently mutating these residues (Y138A/F145A), we demonstrated that the SARS-CoV-2 YF mutant had attenuated replication *in vitro* relative to WT. While the YF mutant had disease similar to WT SARS-CoV-2 *in vivo*, the viral loads in the upper and lower respiratory tracts were significantly decreased. Importantly, we showed that attenuation of the YF mutant is not due to changes in type I IFN sensitivity. Rather, reduced viral replication corresponded to the loss of SG control at early time points post-infection. Overall, our study identifies Y138 and F145 within the NSP3-HVR as critical residues necessary for SARS-CoV-2 inhibition of stress granule formation at the early times post-infection.

**Fig 7:**
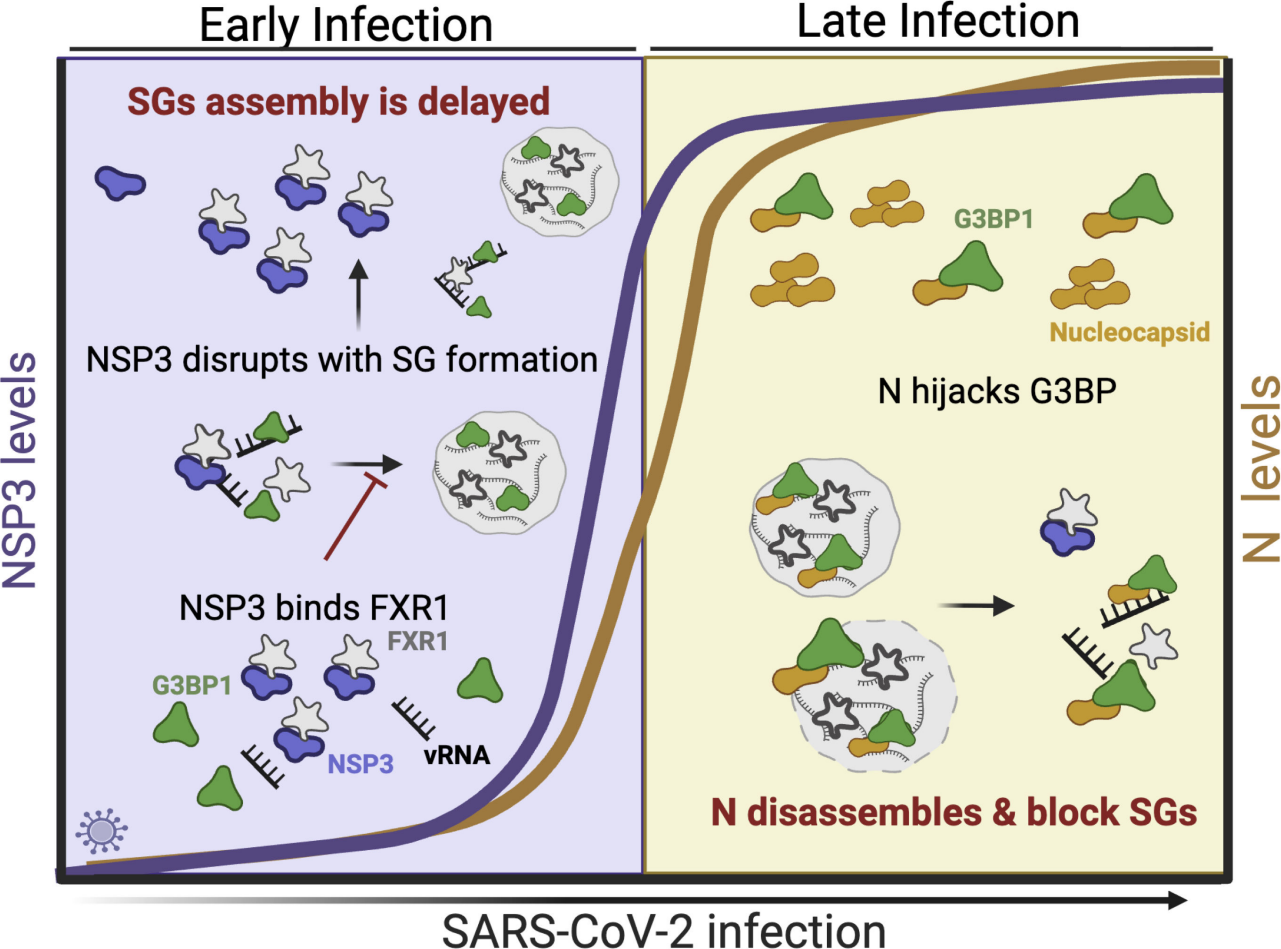
Stress granule formation is restored in YF mutant-infected A549-ACE2 cells during early infection. Early versus late SG regulation. Proposed model of SARS-CoV-2 NSP3 role in SG regulation at early stages of infection. During the early phase of infection, NSP3 HVR binds to FXR1 to disrupt and delay SG formation. At the late stage of infection, SG formation is inhibited by the N protein when expression levels are highest. Overall, NSP3 and N protein work in cooperation to kinetically regulate SG formation during the course of infection.

Stress granule formation is a highly dynamic process and has important implications for host cell functions. In the context of antiviral defense, the contribution of SGs is complex as both translation arrest and induction of innate immune signaling critically impact viral infection. Yet, the strongest evidence for the antiviral role of SGs is the genetic capital viruses use to limit its activation. In the case of SARS-CoV-2, the virus has two distinct viral proteins that contribute to control of SGs: nucleocapsid and NSP3 (Fig. 7). Our group and others have shown that the nucleocapsid of SARS-CoV-2 interacts with G3BP1 to impair SG formation and inhibit innate immune signaling (20, 47). Similarly, our group has previously shown that the HVR of NSP3 binds to FXR1 and disrupts SG formation during the early phase of infection by competing with UBAP2L (30). Here, we define residues Y138 and F145, large aromatic amino acids in the HVR, as the key to FXR1 binding and disrupting interactions with UBAP2L (Fig. 1 and 2). Importantly, these residues are completely conserved in the NSP3 HVR of the surveyed Sarbecoviruses (Fig. 3b) and likely indicate that stress granule antagonism is conserved across the family. Notably, while other CoV families do not have a similar motif in their NSP3 HVR, different CoV proteins have been shown to also antagonize SG function, including MERS-CoV ORF4a, HCoV-OC43 NSP1, SARS-CoV-2 NSP5, and IBV NSP15 (48–51). Together, our results highlight the importance of controlling SG responses during CoV infection. In addition, it indicates that CoVs employ multiple mechanisms to limit stress granule activity.

Stress granule formation is an evolutionarily conserved process found in both mammals and plants to combat stress conditions (9, 52). As such, viruses have also evolved dedicated processes to prevent SG formation. Here, we have shown that two FXR1 binding residues in NSP3 are conserved in SARS-CoV and bat-related Sarbecoviruses. Other work has shown that Y138 is not conserved in MERS, hCoV-229E, or hCoV-OC43 (21); however, F145 is conserved in 229E and OC43, suggesting potential conservation of these amino acids. Specifically, to act as a macromolecular hub for protein assembly needed for efficient viral replication (21), aromatic amino acids such as phenylalanine and tyrosine have been shown to be crucial in mediating LLPS by driving multivalent interactions (53, 54). Therefore, we cannot exclude that these same HVR residues could have an impact on SG formation by other CoVs outside of SARS-CoV-2. Notably, antagonism of SGs by FXR1 is a unique finding. FXRs have been demonstrated to play pivotal roles in viral replication in several RNA viruses and especially defined during alphavirus infection. In the case of New World alphaviruses, VEEV and EEEV have adopted strategies to use FXRs to drive the assembly of viral replication complexes through interaction with their NSP3 hypervariable domain (HVD) (23, 28, 55). Conversely, FXR proteins were recently demonstrated to drive double-membrane vesicle clustering through the same region of NSP3-HVR investigated here (21, 56). However, sequence alignment of Old and New World alphavirus HVDs revealed that there is no presence of conserved tyrosine or phenylalanine throughout the domain. Thus, targeting FXR1 to inhibit SGs by key residues may be a unique feature adopted by CoVs.

In recent years, the link between SG responses and host-antiviral defenses has been complicated by conflicting studies. Studies have argued that SGs amplify antiviral activity by serving as signaling platforms to promote the induction of IFN responses (17, 57). Alternatively, other groups have described a role for SGs as “shock absorbers” to regulate overactivation of innate immunity (58). Others have indicated that loss of SG function had no impact on innate immune responses (19). In this study, we find that loss of early SG control by the YF mutant does not augment sensitivity to type I IFN (Fig. 5a). Similarly, minimal changes in type I IFN or ISGs were observed in the YF mutant as compared to control, suggesting SG induction has limited impact on innate immune signaling (Fig. 5c). However, the accumulation of the N protein and its antagonism of SGs at later times complicates these findings. Importantly, the YF mutant attenuation is not driven by type I IFN responses but rather corresponds to loss of SG control at early times compared to WT (Fig. 6). Overall, the findings indicate activating SG responses may offer a novel route to limit viral infection and pathogenesis.

In addition to defining the key residues in NSP3 driving FXR1 binding, our study also establishes a kinetic profile for SG antagonism by SARS-CoV-2. The N protein has been shown to be a powerful antagonist of SGs by disrupting G3BP1 activity even after SGs have formed (47, 59–62). However, N has several roles during viral infection, including genome packaging, viral assembly, regulation of viral transcription, and modulation of host factors (63–66). These roles are likely controlled by the relative abundance of the N protein and post-translational modifications like phosphorylation (65, 67). Here, we predict that NSP3 and N act at different kinetic times to antagonize SG activity (Fig. 7). We expect N activity against SG to occur later during infection, corresponding to its peak production approximately at 10 hpi (68). In this scenario, SARS-CoV-2 NSP3 functions to disrupt SG at earlier points during infection. At 4 hpi, we detect no difference in SG accumulation between our mutant and WT (Fig. 6d); however, by 6 hpi, we detected a higher fraction of SG-positive cells in our YF-infected group compared to control. This dynamic proposes a kinetic framework by which SARS-CoV-2 NSP3 and N work in cooperation to regulate the SG responses.

Overall, our article identifies aromatic residues Y138 and F145 as critical to the binding of FXR1 and disruption of SG formation during the early parts of SARS-CoV-2 infection. The YF mutant that ablates NSP3 interactions with FXR1 has attenuated growth *in vitro* and *in vivo* that is not governed by augmented type I interferon responses. Instead, we find that the loss of the Y138/F145 results in augmented SG formation at early times and corresponds to attenuated viral replication. Importantly, in combination with the N proteins, our data highlight the genetic capital SARS-CoV-2 invests in controlling SG responses during the course of infection.

## MATERIALS AND METHODS

### Cell lines

African green monkey kidney Vero E6 cells were cultured in high-glucose Dulbecco’s modified Eagle medium (DMEM) (Gibco #11965-092) supplemented with 10% Fetal Clone II (FBS) (Hyclone, #SH30071.03) and 1% antibiotic/antimycotic (anti-anti) (Gibco #5240062). Vero E6 cells expressing human TMPRSS2 were grown in high-glucose DMEM supplemented with 10% FBS, 1% anti-anti, and 1 mg/mL Geneticin (G418) (Gibco, 10131035). Calu-3-2b4 (Calu-3) cells were grown in high-glucose DMEM with 10% defined FBS, 1% anti-anti, and 1 mg/mL sodium pyruvate (1 mM). A549 cells expressing human ACE2 were grown in 10% FBS, 1% anti-anti, and 10 µg/mL Blasticidin S HCL (Gibco, #A1113902).

Vero E6 and Calu-3 cells were provided by Dr. Ralph Barric. A549-ACE2 cells were provided by Dr. Pei Yong Shi. All cell lines were grown at 37°C with 5% CO_2_ and were mycoplasma tested periodically (last negative test in 2024). Single-cell FACS sorting (BD Aria Fusion) was applied on Vero-TMPRSS2 and A549-ACE2 cells to generate TMPRSS2- or ACE2-positive cell populations.

### Viruses

#### Reverse genetic clones

All recombinant viruses were generated using reverse genetics as previously described (31, 32). WT and mutant SARS-CoV-2 sequences are used from the USA-WA1/2020 isolate sequence provided by the World Reference Center for Emerging Viruses and Arboviruses, originally obtained from the US Centers for Disease Control and Prevention, as previously described (69, 70). The NSP3 YF mutant was constructed using standard cloning techniques as previously outlined for our reverse genetics system (31, 32), with virus stocks being amplified on Vero E6-TMPRSS2 to prevent mutations from occurring in furin cleavage sites (71). All viruses were expanded once (P1) for use in studies and titered by focus-forming assay (FFA) and plaque assay (for plaque morphology). All infections and manipulations were performed in a biosafety level 3 laboratory in accordance with approved protocols and use of appropriate personal protective equipment. P1 viral stocks were verified through Sanger sequencing of complementary DNA (cDNA) for mutations in the spike furin cleavage site and the NSP3-HVR for the introduced mutations.

### Biosafety

University of Texas Medical Branch is a registered Research Facility under the Animal Welfare Act. It has a current assurance (A3314-01) with the Office of Laboratory Animal Welfare, in compliance with NIH Policy. Procedures involving infectious SARS-CoV-2 were performed in the Galveston National Laboratory ABSL3 facility.

### *In vitro* infection

#### Viral replication kinetics

Viral replication studies were performed as previously described (30). Briefly, cells (Vero E6, A549-ACE2) were seeded in six-well plates on the day before infection. Calu-3 cells were seeded at high density and allowed to grow to confluency in six-well plates before conducting a growth curve. Cells were infected at the indicated MOI for 45 minutes at 37°C with 5% CO_2_ and every 15 minutes. After absorption, plates were washed three times with PBS, and fresh media were replaced in wells, indicating time 0. Wells were sampled at specified time points. Three technical replicates were collected for each time point, and each experiment was performed twice. Samples were titrated with a focus-forming assay.

For high MOI infections, A549-ACE2 cells were plated in 12-well plates at 5 × 10^5^ cells per well. Viruses were diluted in PBS and added to cells and incubated for 45 minutes at 37°C with 5% CO_2_ and rocked every 15 minutes. Cells were washed three times and replaced with complete DMEM (10% FBS with 1× anti-anti). For each time point, 200 μL of supernatant was collected for viral titers, and cells were lysed for quantitative PCR (qPCR) gene expression analysis with Trizol.

#### Type I IFN assay

For IFN pretreatment, 100 U/mL of recombinant IFN-β (PBL Laboratories) was added to Vero E6 cells 16–18 h prior to infection. Growth curves were conducted as mentioned above.

### *In vivo* infection

#### Animal studies

Three- to four-week male Syrian golden hamsters (HsdHAN:AURA strain) were purchased from Envigo (Indianapolis, IN, USA). For infection, hamsters were intranasally inoculated with 1 × 10^5^ FFU of SARS-CoV-2 WT or YF mutant in a 100 µL volume. Infected animals were weighed and monitored daily for illness over 7 days. Cohorts of five animals per group (including mock-infected animals) were anesthetized with isoflurane, and nasal washes were collected in 400 µL PBS on endpoint days (2, 4, and 7 dpi) for titers. Animals were then humanely euthanized by CO_2_ immediately following nasal washes for lung specimen collection. Lobes were stored in RNAlater solution (Invitrogen) for qPCR analysis and in PBS for titer analysis.

### Focus-forming assay

FFAs were performed as previously described with adaptations (72). Briefly, Vero E6 cells were seeded in 96-well black-walled plates to be 100% confluent the next day. Cell culture supernatants, nasal washes, or homogenized tissues containing SARS-CoV-2 underwent 10-fold serial dilutions in serum-free media. Twenty microliters of diluted and undiluted samples was added to wells and incubated for 45 minutes with rocking every 15 minutes. After absorption, methylcellulose overlay (0.85%) was then added to each well, and cells were incubated for 24 h. The next day, the methylcellulose was removed, and cells were washed three times with PBS and fixed in 10% buffered formalin for 30 minutes at room temperature (rt) to inactivate the virus. For staining, cells were permeabilized and blocked with PBS solution containing 0.1% saponin and 0.1% BSA (perm. buffer) for 30 minutes. Following incubation, α-SARS-CoV-2 nucleocapsid primary antibody (Cell Signaling, 68344) at 1:3,000 in perm. buffer overnight at 4°C. Cells were washed three times with 1× DPBS before incubating with Alexa Fluor 555-conjugated α-mouse secondary antibody (Invitrogen, A28180) at 1:2,000 in perm. buffer for 1 h at rt wrapped in foil. Cells were washed three times with 1× DPBS, and fluorescent foci images were captured using a Cytation 7 cell imaging multi-mode reader (Biotek). Foci were counted using ImageJ free software (NIH).

#### Homogenization

Hamster lungs were infected as described in Animal Studies, and the right inferior lobe was stored in sterile PBS and zirconia beads and kept at −80◦C. Tissues were homogenized using a MagNALyser (Roche Life Science) at 6,000 rpm for a total of 1 minute.

### Real-time qPCR

#### RNA extraction and qPCR

Total RNA from cells was isolated with Direct-zol RNA Miniprep Plus kit (Zymo, #R2072) according to the manufacturer’s protocol. RNA was reverse transcribed to cDNA using the iScript cDNA synthesis kit according to the manufacturer’s instructions (Bio-Rad, 1708891). RT-qPCR was performed on cDNA using the Luna Universal qPCR Master Mix (NEB, #M3003) per manufacturer’s instructions. Fluorescent readings were measured on a Bio-Rad CFX Connect instrument using Bio-Rad CFX Maestro 1.1 (version 4.1.2433.1219). The primers used to amplify host targets were human GAPDH (forward: 5′-TCAAGATCATCAGCAATGCC, reverse: 5′-AAGTTGTCATGGATGACCTTGG); IFIT1 (forward:5′-CCAAGGAGACCCCAGAAACC, reverse: 5′-CGCTACGTGGAGTGAGCTAG); IFIT3 (forward: 5′-AAGAACAAATCAGCCTGGTCAC, reverse: 5′-TCCCTTGAGACACTGTCTTCC); IFNβ (forward: 5′-AGTAGGCGACACTGTTCGTG, reverse: 5′-AGCCTCCCATTCAATTGCCA); OAS1 (forward: 5′-GAGCTCCTGACGGTCTATGC, reverse: 5′-TCATCGTCTGCACTGTTGCT); MDA5 (forward: 5′-AAGCCCACCATCTGATTGGAG, reverse: 5′-CCACTGTGGTAGCGATAAGCAG); and PKR (forward: 5′-GAAGTGGACCTCTACGCTTTGG, reverse: 5′-GATGATGCCATCCCGTAGGTC). Average *C*_*t*_ values of technical replicates were normalized using the ΔΔ*C*_*t*_ method to the housekeeping gene (human GAPDH) and expressed as log_2_ relative fold change.

### Immunofluorescence and microscopy

A549-ACE2 cells were seeded on 96-well black-sided plates 1 day prior to infection. Infections were conducted at the indicated MOI in an inoculum volume of 50 µL volume per well. Incubation was conducted as mentioned in Viral Replication Kinetics. Wells were replaced with fresh cell culture media containing no selective antibiotics. To induce SG formation, wells were replaced with cell culture media containing 1mM sodium arsenite 30 minutes prior to the inactivation time point. Plates were inactivated in 10% buffered formalin at indicated hours post-infection. Antibody staining was adapted from Lamichhane et al. (73). Briefly, fixed cells were permeabilized with 0.2% Triton X-100 in PBS for 10 minutes at rt and blocked with 2% BSA for 1 h. Samples were then incubated with primary antibodies (rabbit anti-G3BP1 [Cell Signaling #61559] and mouse antinucleocapsid [Cell Signaling, 68,344BC]) overnight at 4°C. The next day, cells were washed three times with PBS and incubated with host-specific Alexa Fluor 488 (Invitrogen, A32731) and Alexa Fluor 555 (Invitrogen, A28180) secondary antibodies for 1 h at rt, away from light (73). After the secondary incubation, plates were washed three times, and nuclei were stained with 1 μg/mL Hoecsht 3342 (Thermo Fisher, H1399) according to manufacturer instructions for 15 minutes at rt. Images were captured using a Cytation 7 instrument at ×20 magnification.

### Stress granule quantification

Quantification of SGs was adapted from Dolliver et al. (48). Briefly, the total percentage of G3BP1-positive cells was obtained by counting the total number of cells based on Hoechst staining. First, the total number of nuclei was counted using a self-developed ImageJ macro to automatically threshold and count total cells in an area. At least four areas were imaged in one well (repeated in triplicate) with consistent exposure setting for all images. Cells containing at least one granule were counted as SG+ using the multi-point tool in ImageJ (48). A minimum of 50 cells per area was counted for each condition in each experiment. Images were analyzed using ImageJ 1.54p Fiji. For robust image analysis, standardized image acquisition practices were followed. Low to moderate cell densities were imaged to prevent negative impacts on cell counting and segmentation. Image acquisition was carried out using optimal constant exposure settings for each experiment. Background signal subtraction was performed using negative controls in ImageJ. The QuickFigures plugin for ImageJ was used to prepare immunofluorescence panels. (74)

### Structure modeling

Structural models were generated to visualize FXR1 and NSP3 residue interactions using AlphaFold as previously described (30). Models were generated using PyMOL (version 3.10) for WA-1 NSP3 and human FXR1. Residues Y138 and F145 are highlighted and mutated to visualize alanine mutations in YF residues of NSP3.

### Immunoprecipitations

HeLa cells were transfected with 2 μg DNA and 2μL Jet Optimus reagent overnight. Cell pellets were collected after 24 h and lysed in 400 μL lysis buffer (100 mM NaCl, 50 mM Tris, pH 7.4, 0.1% NP40, 0.2% Triton-100, and 1 mM DTT) supplemented with protease (complete mini EDTA free, Roche) and phosphatase inhibitor tablets (Roche) for 45 minutes on ice. Lysates were cleared at 20,000 × *g* at 4°C for 1 h. Cleared lysates were incubated with 10 μL pre-equilibrated GFP-trap beads for 1 h at 4°C on a rotor wheel. Following three washes with 1 mL wash buffer (150 mM NaCl, 50 mM Tris, pH 7.4, 0.05% NP40, 5% glycerol, and 1 mM DTT), the samples were eluted with 25 μL 2× LSB (Thermo) and processed for Western blot using the following antibodies: FXR1 mouse (Santa Cruz, 374148) and GFP rabbit (made in house against GFP).

### Isothermal calorimetry

ITC experiments were set up as described in reference 30. The following peptides, purchased from Peptide 2.0 Inc (Chantilly, VA, USA) were used:

NSP3 WT: QYEYGTEDDYQGKPLEFGATSW

NSP3 YF/AA: QYEYGTEDD**A**QGKPLE**A**GATSW.

## Data Availability

The raw data of this study are available from the corresponding author upon request.
